# DNA Barcoding Identifies Unknown Females and Larvae of *Fannia* R.-D. (Diptera: Fanniidae) from Carrion Succession Experiment and Case Report

**DOI:** 10.3390/insects12050381

**Published:** 2021-04-23

**Authors:** Andrzej Grzywacz, Mateusz Jarmusz, Kinga Walczak, Rafał Skowronek, Nikolas P. Johnston, Krzysztof Szpila

**Affiliations:** 1 Department of Ecology and Biogeography, Faculty of Biological and Veterinary Sciences, Nicolaus Copernicus University in Toruń, 87-100 Toruń, Poland; walczak.kinga00@gmail.com (K.W.); nikolaspjohoston@gmail.com (N.P.J.); szpila@umk.pl (K.S.); 2 Department of Animal Taxonomy and Ecology, Faculty of Biology, Adam Mickiewicz University in Poznań, 61-712 Poznań, Poland; mat.jarmusz@gmail.com; 3 Department of Forensic Medicine and Forensic Toxicology, Faculty of Medical Sciences in Katowice, Medical University of Silesia in Katowice, 40-055 Katowice, Poland; rafal-skowronek@wp.pl

**Keywords:** Fanniidae, larval morphology, forensic entomology, human cadaver

## Abstract

**Simple Summary:**

Insects are frequently attracted to animal and human cadavers, usually in large numbers. The practice of forensic entomology can utilize information regarding the identity and characteristics of insect assemblages on dead organisms to determine the time elapsed since death occurred. However, for insects to be used for forensic applications it is essential that species are identified correctly. Imprecise identification not only affects the forensic utility of insects but also results in an incomplete image of necrophagous entomofauna in general. The present state of knowledge on morphological diversity and taxonomy of necrophagous insects is still incomplete and identification of immature and female forms can be extremely difficult. In this study, we utilized molecular identification methods to link conspecific sexes and developmental stages of forensically important flies. We identified larvae and females of flies collected from animal and human cadavers which otherwise were morphologically unidentifiable. The present study fills a gap in taxonomy of flies and provides data facilitating application of new species as forensic indicators.

**Abstract:**

Application of available keys to European Fanniidae did not facilitate unequivocal species identification for third instar larvae and females of *Fannia* Robineau-Desvoidy, 1830 collected during a study of arthropod succession on pig carrion. To link these samples to known species, we took the advantage of molecular identification methods and compared newly obtained cytochrome oxidase subunit I (COI) barcode sequences against sequences deposited in reference databases. As an outcome of the results obtained, we describe for the first time a third instar larva of *Fannia nigra* Malloch, 1910 and *Fannia pallitibia* (Rondani, 1866) and a female of *Fannia collini* d’Assis-Fonseca, 1966. We provide combinations of characters allowing for discrimination of described insects from other Fanniidae. We provide an update for the key by Rozkošný et al. 1997, which allows differentiation between females of *F. collini* and other species of Fanniidae. Additionally, we provide a case of a human cadaver discovered in Southern Poland and insect fauna associated with it as the first report of *F. nigra* larvae developing on a human body.

## 1. Introduction

Fanniidae is one of the dipteran families that are attracted to and develop in decomposing animal carrion and human bodies [1,2,3,4,5]. Fanniids can be found at various stages of decomposition and are also known for their ability to exploit both buried remains [6,7] and those restricted to indoor conditions [8,9]. Under certain circumstances, species of Fanniidae may be utilized as forensic indicators [4,5,8,9]. However, the broad application of Fanniidae for medico-legal purposes is inhibited by the general difficulty of species identification in this family [10] and the absence of information linking females and immature stages to the more rigorously studied adult males.

In many species the linkage of conspecific males and females is hindered by sexual dimorphism, especially differences in coloration and leg ornamentation between males and females of the same species. Morphological characters that are diagnostic in males, such as the body color, specific structures on legs and genital structures, frequently vary or are uninformative for the identification of females. Further complicating the identification of female fanniids are two issues; firstly, that some species are described only from males, and secondly females of closely related fanniids (when described) are frequently discriminated based only on a few vague characters, such as minor differences in body coloration or the number or size of fine setae. This is of particular detriment to the forensic utility of fanniids as the majority of adult specimens collected from carrion succession experiments or crime scene are females [1,2,4], and therefore in some cases the adult females are impossible to identify based on morphology alone.

The differentiation of larvae and puparia of Fanniidae from other forensically relevant dipteran families is relatively straightforward due to the characteristic dorso-ventrally flattened body, thoracic and abdominal segments equipped with fleshy projections and posterior spiracles raised on stalks. The pattern of fleshy processes on thoracic and abdominal segments is often species-specific [11,12]. However, morphological interpretations of some character states vary between various authors and require revision, without which accurate discrimination between closely related species will be challenging [13]. Furthermore, immature stages have only been described for several species attracted to carrion and dead bodies, and therefore it is likely that there is much morphological diversity among larvae yet to be discovered.

Animal carrion and dead human bodies have been observed to attract more than 50 species of fanniids worldwide [1,2,3,14,15,16], with 15 species confirmed as developing on cadavers [3,4,16,17,18,19,20,21,22,23,24,25]. However, due to taxonomic issues and difficulties in obtaining accurate species identifications, most studies refer only to a few common species of *Fannia* Robineau-Desvoidy, 1830 (e.g., *Fannia canicularis* (Linnaeus, 1761), *Fannia manicata* (Meigen, 1826), *Fannia pusio* (Wiedemann, 1830) and *Fannia scalaris* (Fabricius, 1794)) [23,26]. The overrepresentation of these species in the literature provides an incomplete representation of carrion entomofauna, which falsely implies that the most frequent and numerous elements of carrion fanniid assemblages are the commonly identified species mentioned above.

During a study on insect succession on pig carrion, we found two distinct types of third instar larvae, hereafter *Fannia* sp. 1 and *Fannia* sp. 2, and females, hereafter *Fannia* sp. 3, which were not morphologically linked to the commonly encountered carrion-breeding species of *Fannia* [11,12,27,28]. These unidentified *Fannia* spp. outnumbered many other necrophagous species [16]. Furthermore, several specimens corresponding with *Fannia* sp. 1 have also been collected from a dead human body in Southern Poland as part of a forensic investigation.

Based on these findings, we hypothesized: (1) *Fannia* sp. 1 and *Fannia* sp. 2 represent two species which are not known from third instar larvae, (2) *Fannia* sp. 3 represents a species unknown from the female sex and (3) either *Fannia* sp. 1 or *Fannia* sp. 2 is conspecific with *Fannia* sp. 3. To validate our hypotheses and assist in identifying unknown *Fannia* spp. we utilized DNA barcoding. First, we obtained cytochrome oxidase subunit I (COI) barcode sequences, and secondly we compared those against sequences deposited in reference databases. 

## 2. Materials and Methods

### 2.1. Sampling

Materials for this study were collected during a field study of arthropod succession on pig carrion, conducted in Central Poland and from a human cadaver discovered in Southern Poland. The carrion succession experiment was carried out in spring, summer and autumn of 2012 and 2013. Four carcasses were used in every study season (24 pig carcasses total). Comprehensive description of the experimental design and field protocol of the carrion succession experiment can be found in Jarmusz & Bajerlein 2019 and Jarmusz et al. 2020 [16,29]. Insects have been identified using the keys of Rozkošný et al. [27] and Chillcott [28] for immature stages. For morphological examination, larvae were cleaned with a fine brush and anterior body parts were slide mounted in Hoyer’s medium, using concave slides. Images were taken with a Leica DFC450 C digital camera mounted on a Leica DM2500 LED microscope (Leica Camera AG, Germany). Terminology follows Courtney et al. [30] for the general morphology. For family-specific structures, particularly for the processes covering body segments, we are following Lyneborg [12] and Grzywacz et al. [20]. Voucher specimens have been deposited in the collection of the Department of Ecology and Biogeography, Nicolaus Copernicus University in Toruń.

### 2.2. DNA Extraction, Amplification and Sequencing

Total genomic DNA was isolated from thoracic muscles in females and thoracic and abdominal tissues in larvae using a DNeasy Blood & Tissue Kit (Qiagen, Valencia, CA, USA) following the manufacturer’s protocol. Isolated DNA was quantified with a Qubit 3.0 fluorometer using dsDNA High Sensitivity Assay Kit (Life Technologies, Inc., Carlsbad, CA, USA) following the manufacturer’s instructions. To obtain the COI barcode region, we used the primers TY-J-1460 and C1-N-2191 [31,32]. We performed a standard 25-µl PCR for each sample using 1 × PCR buffer, 0.2 mM dNTPs, 0.2 µM of each primer, 2 mM MgCl_2_, 1 U of Taq DNA polymerase (Qiagen) and 1–2 µL of the DNA template. The PCR cycles were as follows: 94 °C for 2 min, 30 cycles 94 °C for 30 s, 50 °C for 30 s and 70 °C for 45 s, followed by a final extension at 70 °C for 10 min.

The PCR products were electrophoresed in a 1% agarose gel, stained with GelRed (Biotium, Darmstadt, Germany) and photographed with a gel documentation system. For sequencing, we only used samples without obvious polymorphisms (multiple bands from a single PCR product). The PCR products were purified with AMPure XP (Beckman Coulter, Carlsbad, CA, USA) (1 × ratio of beads to sample volume). Purified products were re-suspended in TE buffer and the DNA yield was measured using a Qubit 3.0 fluorometer and 2100 Bioanalyzer with the High Sensitivity DNA Analysis kit. Cycle sequencing reactions were carried out using the PCR product (5–20 ng/μL of template DNA) and fluorescent Big Dye terminators (Applied Biosystems, San Francisco, CA, USA). Final products of sequencing were resolved using an automated DNA sequencer at the Laboratory of Molecular Biology Techniques, UAM (Poznań, Poland). Both forward and reverse strands were edited and then assembled using SeqMan II ver. 4.0 (DNASTAR, Lasergene, Madison, WI, USA).

### 2.3. Sequence Alignment and Data Analysis

Obtained sequences were identified by comparison to sequences available in the NCBI database (National Center for Biotechnology Information, Bethesda, MD, USA) using the Basic Local Alignment Search Tool (BLAST) and BOLD v4 (Barcode of Life Data Systems) using BOLD Identification System [33]. All COI barcode sequences available for *Fannia*, including library of COI reference sequences available for forensically relevant Fanniidae [10], have been downloaded from BOLD database and supplemented with newly obtained data. DNA sequences were aligned using MAFFT v7 [34] and visually inspected and trimmed to a 658-bp long barcode fragment in Seaview 4.4.0 [35]. For the graphical presentation of our data, we performed Neighbor Joining (NJ) phylogenetic analysis using the pairwise distance with 1000 bootstrap replications. We pruned from the NJ analysis sequences not assigned to species and when multiple sequences were available for a species, we used up to 20 randomly selected sequences.

## 3. Case Report

On 14th October 2016, at noon, the corpse of a woman was discovered in a forest near the DK86 road in Southern Poland. The body was in a state of active decomposition except the head, where signs of advanced decomposition were observed. The body was naked, and clothes were scattered around it. No signs of unnatural death were observed. The deceased was schizophrenic, in the last period of her life she stopped taking medications and showed psychotic symptoms. According to the findings of the investigation, the woman was last seen alive on 10th September. Entomological material was collected during the inspection of the crime scene. Larvae and pupae of Diptera were immediately preserved in 96% ethanol. Entomological material was identified according to keys of Szpila [36], Fremdt et al. [37], Martín-Vega et al. [38] and Rozkošný et al. [27]. Preimaginal stages of the following species were recorded: third instar larvae of *Calliphora vomitoria* (Linnaeus, 1758), third instar larvae and pupae of *Chrysomya albiceps* (Wiedemann, 1819), third instar larvae of *Fannia* sp. 1 and third instar larvae of *Stearibia nigriceps* (Meigen, 1826). The oldest developmental stages noticed on the corpses were pupae of the *Ch. albiceps*. However, the temperatures in the area of the crime scene between when the deceased was last seen alive and the discovery of the corpse, were low (average 11.1 °C) and close to or below the lower developmental threshold reported for this species [39,40]. Using thermal data provided by a prosecutor, we estimated that the minimum time necessary for pupariation of *Ch. albiceps* was considerably longer than the time the deceased person was missing. Analysis of the *C. vomitoria* larvae gave minimum PMI of 17–18 days before the body was found, before 28th September 2016. The investigation was legally discontinued, as no evidence was found that indicated criminal activity had contributed to the woman’s death.

## 4. Results

### 4.1. Molecular Data and Identification

We successfully amplified the full 658bp COI barcode sequence for two specimens of *Fannia* sp. 1, and two specimens of *Fannia* sp. 3. Due to poor quality material, it was only possible to amplify a short 311 bp fragment for *Fannia* sp. 2. All sequences can be accessed in GenBank through the following accession numbers: MW670438 (Fannia_sp_1_1), MW670439 (Fannia_sp_1_2), MW670440 (Fannia_sp_2), MW670441 (Fannia_sp_3_1) and MW670442 (Fannia_sp_3_2). The newly sequenced COI barcodes were then supplemented with additional 4421 COI barcode sequences, representing 67 *Fannia* species, retrieved from the online databases BOLD. Among those sequences 114 have not been assigned to species or recognized as uncertain identifications.

Comparison of COI barcodes for *Fannia* sp. 1, sp. 2 and sp. 3 with COI data deposited in BOLD and GenBank, enabled identification of these unknown specimens. Sequences obtained for *Fannia* sp. 1 (Fannia_sp_1_1 and Fannia_sp_1_2) were identified as *Fannia nigra* Malloch, 1910, based on 100% similarity with sequences provided from a male (KY511202) and a female (KY511203) of *F. nigra* by Grzywacz et al. [10].

Sequences obtained for *Fannia* sp. 3 (Fannia_sp_3_1 and Fannia_sp_3_2) were identified as *Fannia collini* d’Assis-Fonseca, 1966, based on 99.85% similarity with sequences obtained from male specimens (KY511166, KY511167, KY511168) of *F. collini* by Grzywacz et al. [10].

The sequence obtained for *Fannia* sp. 2 (Fannia_sp_2) was identified as *Fannia pallitibia* (Rondani, 1866) based on 100% similarity with an unpublished sequence assigned to *F. pallitibia* and available in BOLD.

**Figure 1:**
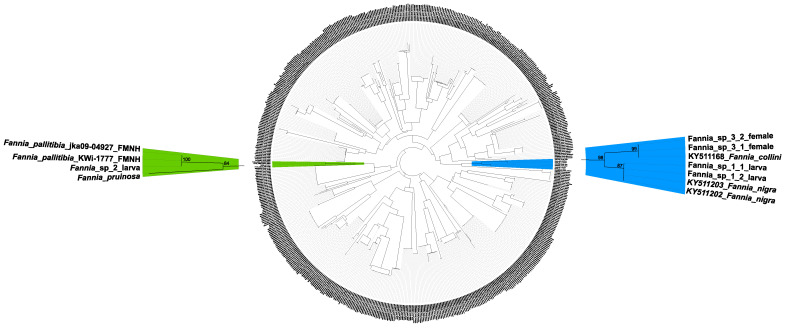
Neighbor joining phylogenetic analysis of 596 COI barcode sequences representing 67 species of *Fannia* retrieved from BOLD and GenBank supplemented with six newly obtained sequences, including three query morpho-species and *Fannia pruinosa*. Values above nodes indicate support for sequence clusters obtained from 1000 bootstrap replications.

To validate obtained identifications, the COI dataset retrieved from BOLD was supplemented with two additional sequences of *F. pallitibia* (voucher specimens jka09-04927 and KWi-1777 accessible through the Finnish Museum of Natural History (FMNH)) and a newly obtained sequence of *Fannia pruinosa* (Meigen, 1826), a species closely related with *F. pallitibia*. COI barcode sequences, referring to unidentified *Fannia* sp. 1, 2 and 3, were compared against the combined dataset using SpeciesIdentifier v1.8 [41]. This approach confirmed identifications obtained from the BOLD Identification System. Fannia_sp_2 sequence was found 100% similar with *F. pallitibia* (jka09-04927). Additionally, phylogenetic analysis confirmed those identifications (Figure 1), and sequences Fannia_sp_1_1 and Fannia_sp_1_2 clustered with those referring to *F. nigra* (BS = 87%), Fannia_sp_2 with *F. pallitibia* (BS = 100%) and sequences Fannia_sp_3_1 and Fannia_sp_3_2 with *F. collini* (BS = 99%).

### 4.2. Fannia nigra Malloch, 1910

**Figure 2:**
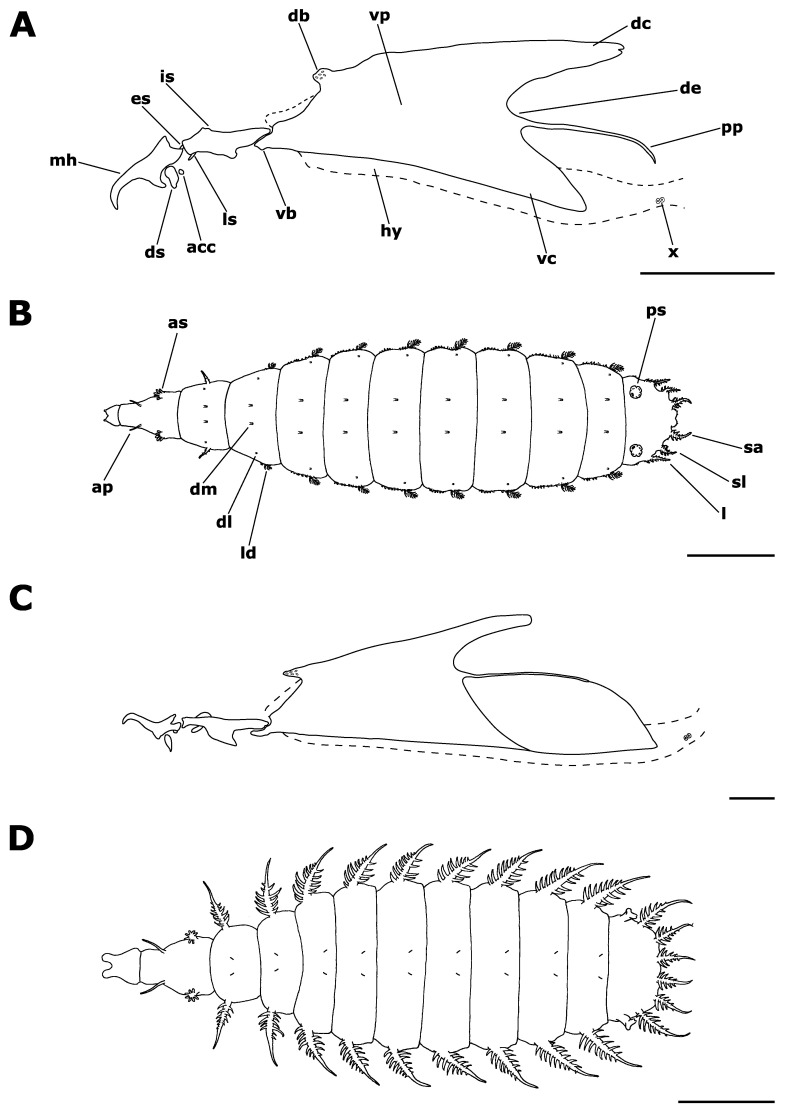
Third instar larvae of *Fannia*: (**A**) cephaloskeleton, *Fannia nigra*; (**B**) larva in dorsal view, *Fannia nigra*; (**C**) cephaloskeleton, *Fannia pallitibia*; (**D**) larva in dorsal view, *Fannia pallitibia*. Scale bare 100 µm (**A**,**C**) and 1 mm (**B**,**D**). Abbreviations: acc, accessory stomal sclerite; ap, anterior process; as, anterior spiracle; db, dorsal bridge; dc, dorsal cornu, de, dorsal extension; dl, dorsolateral process; dm, dorsomedian process; ds, dental sclerite; es, epistomal sclerite; hy, hypopharynx; is, intermediate sclerite; l, lateral process; ld, laterodorsal process; ls, labial sclerite; mh, mouthhook; pp, posterior projection; ps, posterior spiracle; sa, subapical process; sl, sublateral process; vb, ventral bridge; vc, ventral cornu; vp, vertical plate; x, sensory organ X.

**Figure 3:**
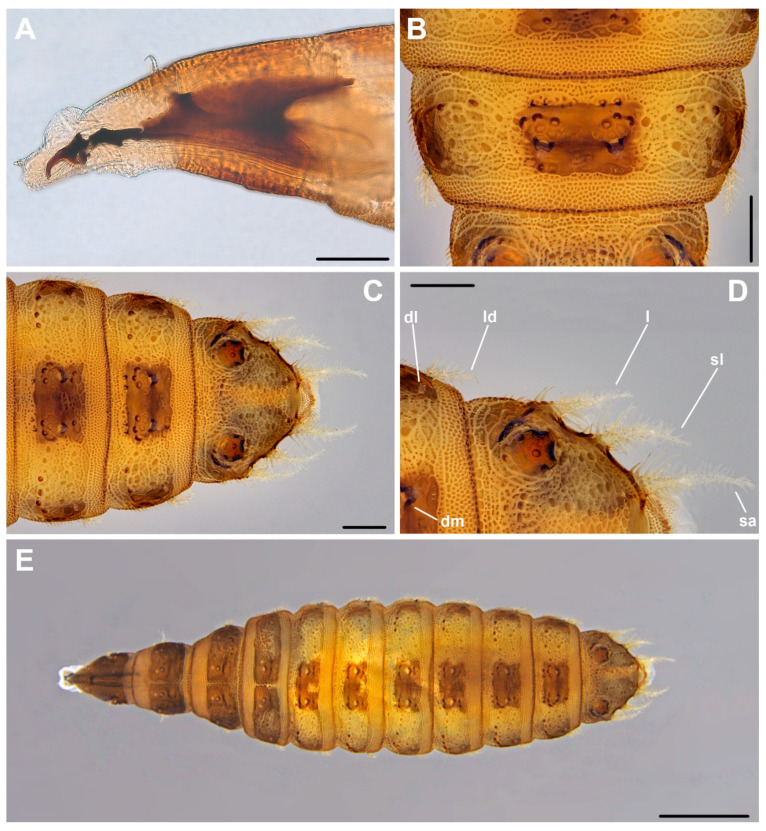
Third instar larva of *Fannia nigra*: (**A**) anterior body end with cephaloskeleton; (**B**) seventh abdominal segment, dorsal view; (**C**) posterior body end, dorsal view; (**D**) seventh abdominal segment and anal division, dorsal view; (**E**) larva in dorsal view. Scale bar 100 µm (**A**), 250 µm (**B**–**D**) and 1 mm (**E**). Abbreviations: dl, dorsolateral process; dm, dorsomedian process; l, lateral process; ld, laterodorsal process; sa, subapical process; sl, sublateral process.

In Figure 2A,B and Figure 3. Material examined. Third instar larvae from pig carrion: 2, 23 V 2012; 2, 2 VI 2012; 2, 5 VI 2012; 6, 7 VI 2012; 1, 11 VI 2012; 1, 13 VI 2012; 2, 15 VI 2012; 2, 22 VI 2012; 1, 25 VI 2012; 3, 2 VII 2012; 2, 6 VII 2012; 2, 19 VII 2012; 2, 2 VIII 2012; 1, 4 VIII 2012; 1, 7 VIII 2012; 4, 10 VIII 2012; 2, 12 VIII 2012; 2, 23 VIII 2012; 1, 27 VIII 2012; 2, 3 IX 2012; 4, 11 IX 2012; 1, 5 X 2012; 1, 11 X 2012; 2, 14 X 2012; 2, 15 X 2012; 2, 19 X 2012; 10, 23 X 2012; 20, 25 X 2012; 18, 27 X 2012; 19, 3 XI 2012; 36, 7 XI 2012; 6, 11 XI 2012; 34, 15 XI 2012; 7, 26 XI 2012; 17, 3 XII 2012; 1, 26 V 2013; 1, 29 V 2013; 1, 30 V 2013; 2, 1 VI 2013; 1, 2 VI 2013; 10, 3 VI 2013; 2, 4 VI 2013; 2, 5 VI 2013; 13, 6 VI 2013; 6, 7 VI 2013; 7, 9 VI 2013; 11, 11 VI 2013; 4, 13 VI 2013; 5, 15 VI 2013; 1, 17 VI 2013; 25, 21 VI 2013; 157, 26 VI 2013; 42, 30 VI 2013; 24, 4 VII 2013; 2, 11 VII 2013; 1, 18 VII 2013;1, 6 VIII 2013; 6, 9 VIII 2013; 3, 10 VIII 2013; 1, 12 VIII 2013; 1, 14 VIII 2013; 1, 16 VIII 2013; 2, 18 VIII 2013; 4, 20 VIII 2013; 4, 22 VIII 2013; 1, 30 VIII 2013; 14, 4 IX 2013; 3, 11 IX 2013; 5, 18 IX 2013; 2, 22 X 2013; 1, 23 X 2013; 4, 25 X 2013; 4, 28 X 2013; 9, 30 X 2013; 3, 1 XI 2013; 14, 3 XI 2013; 10, 5 XI 2013; 20, 7 XI 2013; 19, 9 XI 2013; 13, 12 XI 2013; 8, 15 XI 2013; 2, 18 XI 2013; 23, 25 XI 2013; 12, 2 XII 2013; 14, 16 XII 2013; 3, 12 II 2014; hornbeam-oak forest, Biedrusko, Poland, M. Jarmusz leg. Third instar larvae from a human cadaver: 8, 14 X 2016; Sarnów near Będzin, Poland.

Third instar larva dorso-ventrally flattened; young larvae creamy-white, mature larvae yellowish to brownish.

Cephaloskeleton. Cephaloskeleton distinctly chitinized (Figure 3A). Mouthhook (*mh*) with apical part downcurved and basal part of *mh* anteriorly equipped with a distinct, ventrolateral extension (Figure 2A). Posterodorsal and posteroventral angles of *mh* drawn out into pointed processes. Paired dental sclerites (*ds*) and accessory stomal sclerites (*acc*) below the basal part of *mh*. Intermediate sclerite (*is*) elongated and H-shaped, with a broad crossbeam. In the lateral view crossbeam visible as a distinct process directed postero-ventrally. Epistomal sclerite (*es*) lies freely between anterior arms of *is*, above the crossbeam; *es* not visible in lateral view. A pair of labial sclerites (*ls*) present below the crossbeam. Basal sclerite (*bs*) consists of very broad vertical plate (*vp*), dorsal cornu (*dc*) and ventral cornu (*vc*). Parastomal bar not developed. Both *vp* connected antero-dorsally by a perforated dorsal bridge (*db*) and antero-ventrally by a ventral bridge (*vb*). Anterior margin of *vp*, between *vb* and *db*, equipped with lighter portion of sclerotisation (possibly additional patch of sclerotisation increasing with larval age). The length of *dc* is about the same or slightly longer than *vc*. Dorsal extension (*de*) of *vc* equipped with well sclerotized and down-curved posterior projection (*pp*). Vertical plate (*vp*) and *vc* are connected below for their entire length by a weakly chitinized hypopharynx, bearing longitudinal ridges. In lower posterior part of *vc*, a sensory organ X (*x*), equipped with paired sensilla is present.

Anterior and posterior spiracles. Anterior spiracles (*as*) fan-shaped, with about 6–9 relatively long lobes, each. Posterior spiracles (*ps*) well separated from lateral margin of anal division (*ad*) (Figure 3C). Posterior spiracles (*ps*) slightly raised on stalks above the surface of *ad* (Figure 3D). Each of the three respiratory slits (*rs*) placed on a short undistinguished finger-like lobe. The plate of each of *ps* without distinct spiracular tufts (*st*), which are probably reduced to a form of single sensillum or complex of trichoid sensilla.

Pattern of processes. Thoracic (T1–3) and abdominal (A1–7) segments equipped with relatively short or inconspicuous projections (Figure 2B and Figure 3E). On T1, two pairs of lateral processes (*lp*), each in form of a cluster of short prominences, present below *as*. Anterior margin of T1 dorsal surface with a pair of forwardly directed, short anterior processes (*ap*). Dorsomedian (*dm*), dorsolateral (*dl*), laterodorsal (*ld*), lateroventral (*lv*), ventrolateral (*vl*) and ventromedian (*vm*) processes present from the second thoracic (T2) to the seventh abdominal (A7) segment. Dorsomedians (*dm*) weakly developed, and present in form of a basal ring equipped with a cluster of minute projections (Figure 3D). Dorsolateral (*dl*) present as a circle of minute projections. Both *dm* and *dl* of similar size, but *dl* devoid of the basal ring. On the thoracic segments *dl* are situated in the anterior part of each segment, between *dm* and *ld*, but when on abdominal segments *dl* are placed on the posterior part of each segment, close to the base of *ld*. The strongest processes on thoracic and abdominal segments are *ld* (Figure 2B and Figure 3B). On T2 *ld* form a process with minute lateral projections. On remaining segments, T3–A7, *ld* in form a short process equipped with lateral projections. On T2 *ld* directed anteriorly, on remaining segments *ld* directed posteriorly. Rows of minute, simple projections precede abdominal *ld* (Figure 3B). Thoracic *lv* form a circle of minute projections placed either on the anterior part of the T2 or in the middle part of the T3. On T3 *lv* followed by a short row of minute projections. Abdominal *lv* present as very short stalks with lateral projection, placed on the posterior part of each segment, and preceded with a row of minute projections. Thoracic *vm* present on the anterior part of segments, and abdominal *vm* on the posterior margin A1–7, all have a form of a pair of circles of minute projections. Second pair of *vm* indistinct. Ventrolaterals (*vl*) in form of small, yet distinct tufts of projections and are well marked on T2–A7 and *ad*.

Sublateral processes (*sl*) and lateral (*l*) processes on anal division of similar length (Figure 3D). Subapical (*sa*) processes appear somewhat, yet not distinctly longer. All three pairs of processes equipped with distinct lateral projections, mostly simple, not bifurcated. Lateral projections are longest in basal half of each process and shorten towards apical part of process.

Integumental sculpture. Thoracic and abdominal segments with a pattern of small, discrete elements, some of which grouped in irregular polygons (Figure 3B). Elements grouped in polygons separated by fine lines, while others widely spaced. Dorsally, integumental pattern is more or less obscured by uniformly smooth, dark areas, arranged in transverse lines. Anterior margin of T3–A7 dorsally covered with smooth polygonal plates arranged in a single row. Posterior margin of T1–A7 dorsally and anterior margin of T1–A1 ventrally, covered with fine, dense, convex elements devoid of projections. Anterior margin of A2–7 with transverse line of scale-like elements ventrally, arranged in groups of at least three elements.

### 4.3. Fannia pallitibia (Rondani, 1866)

**Figure 4:**
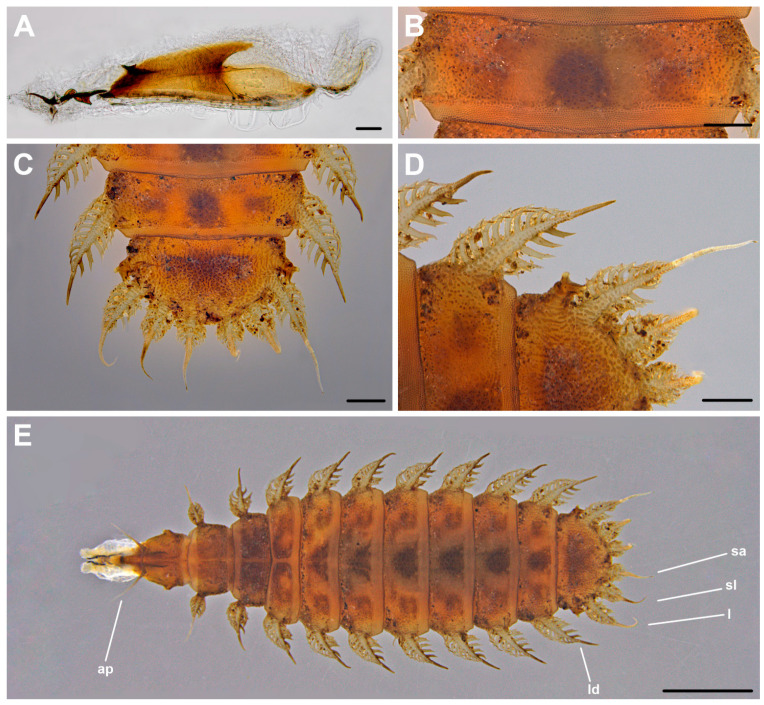
Third instar larva of *Fannia pallitibia*: (**A**) anterior body end with cephaloskeleton; (**B**) sixth abdominal segment, dorsal view; (**C**) posterior body end, dorsal view; (**D**) seventh abdominal segment and anal division, dorsal view; (**E**) larva in dorsal view. Scale bar 100 µm (**A**), 250 µm (**B**–**D**) and 1 mm (**E**). Abbreviations: ap, anterior process; l, lateral process; ld, laterodorsal process; sa, subapical process; sl, sublateral process.

In Figure 2C,D and Figure 4. Material examined. Third instar larvae from pig carrion: 1, 19 VII 2012; 3, 7 XI 2013; hornbeam-oak forest, Biedrusko, Poland, M. Jarmusz leg.

Third instar larva dorso-ventrally flattened, young larvae creamy-white, mature larvae yellowish to brownish.

Cephaloskeleton. Cephaloskeleton well chitinised, with postero-ventral part of basal sclerite transparent (Figure 4A). Apical part of *mh* directed dorsally, with hook-like distal part (Figure 2C). Basal part of *mh* distinctly bifurcated posteriorly, anteriorly equipped with an indistinct ventrolateral extension. Paired scale-shaped *ds* and minute *acc* present below basal part of *mh*. Intermediate sclerite (*is*) long, yet in general appearance not enlarged, arms relatively narrow. In the lateral view crossbeam of *is* distinctly elongated postero-ventrally. Epistomal sclerite (*es*) well visible in lateral view; a pair of labial sclerites (*ls*) present below the crossbeam. Basal sclerite (*bs*) consists of very broad, vertical plate (*vp*), dorsal cornu (*dc*) and ventral cornu (*vc*). Parastomal bar not developed. Both *vp* connected antero-dorsally by a perforated dorsal bridge (*db*) and antero-ventrally by a ventral bridge (*vb*). The anterior margin of *vp*, between *vb* and *db*, equipped with lighter portion of sclerotisation (possibly additional patch of sclerotisation increasing with larval age). The length of *dc* is the same as *vc*. Dorsal extension (*de*) of *vc* equipped with indistinctly sclerotized, yet well visible, down-curved posterior projection (*pp*). Ventral cornu (*vc*) generally smooth, extends posteriorly with weakly pigmented scale-like element. Upper margin of this element is delimited by *pp* and lower margin by a hypopharynx. Through the entire length pairs of *vp* and *vc* are connected below by a weakly chitinized hypopharynx, bearing longitudinal ridges. In lower posterior part of *vc*, a sensory organ X (*x*), is present and equipped with paired sensilla.

Anterior and posterior spiracles. Anterior spiracles (*as*) small, rounded, equipped with about 6 indistinct lobes. Posterior spiracles (*ps*) well separated from each other, placed on the lateral margin of anal division (*ad*) (Figure 4C). Posterior spiracles raised on distinct, apically bifurcated stalks (Figure 4D). Two respiratory slits (*rs*) placed on single, distinct finger-like lobe, the third *rs*, the outer one, placed on a second finger-like lobe. Spiracular tufts (*st*) indistinct, most likely reduced to a form of single sensillum or complex of trichoid sensilla.

Pattern of processes. Thoracic (T1–3) and abdominal (A1–7) segments with long laterodorsals (*ld*), remaining processes inconspicuous or absent (Figure 2D and Figure 3E). Processes close to anterior spiracles (*as*) not present. Anterior margin of T1 with a pair of forwardly directed, long anterior processes (*ap*). Dorsomedians (*dm*) weakly developed and in form of minute filiform processes. Thoracic *dm* located in the mid part of each segment and directed anteriorly. Abdominal *dm* present on the posterior part of each segment and directed posteriorly and. Dorsolaterals (*dl*) absent or indistinguishable under the stereomicroscope (Figure 4D). Laterodorsals (*ld*) strong. Thoracic *ld* equipped with long, bifurcated lateral projections in the basal half, while abdominal *ld* equipped with long projections in the basal two thirds. Thoracic *ld* not directed posteriorly, abdominal *ld* directed posteriorly. Lateroventrals (*lv*) in a form of a cone, built from a circle of minute projections. First pair of *lv* (on T2) minute and placed close to the base of *ld*. Thoracic *lv* smaller than abdominal *lv*. One pair of ventromedians (*vm*), in form of a circle of minute projections, present on each thoracic segment. Second pair of *vm* indistinct. Each abdominal segment carries two or three pairs of *vm* in form of a sensillum on low callus. Thoracic *vm* present on the anterior part of segments, and abdominal *vm* on the posterior margin of each segment. Thoracic ventrolaterals (*vl*) very small, in form of circles of minute projections. Abdominal *vl* small, yet well visible, present as cones composed from minute projections. Sublateral processes (*sl*), lateral (*l*) and subapical (*sa*) processes long and of similar length (Figure 2D and Figure 4C). Three pairs of processes on anal division equipped with distinct, bifurcated lateral projections in basal half.

Integumental sculpture. Thoracic and abdominal segments with a pattern of small, discrete elements (Figure 4B). Elements on outer margins of thoracic and abdominal segments and on the basal part of laterodorsals (*ld*) wart-like in shape. In the middle part of segment, towards lateral margins, elements are grouped in irregular polygons. Polygons separated by fine lines, while other elements are widely spaced. Dorsally, integumental pattern more or less obscured by uniformly smooth, darker areas (Figure 4C,E). Anterior margin of T3–A7 dorsally covered with smooth polygonal plates arranged in a single row. Posterior margin of T1–A7 dorsally, and anterior margin of T1–A1 ventrally, covered with fine, dense, convex elements devoid of projections.

### 4.4. Fannia collini d’Assis-Fonseca, 1966 

**Figure 5:**
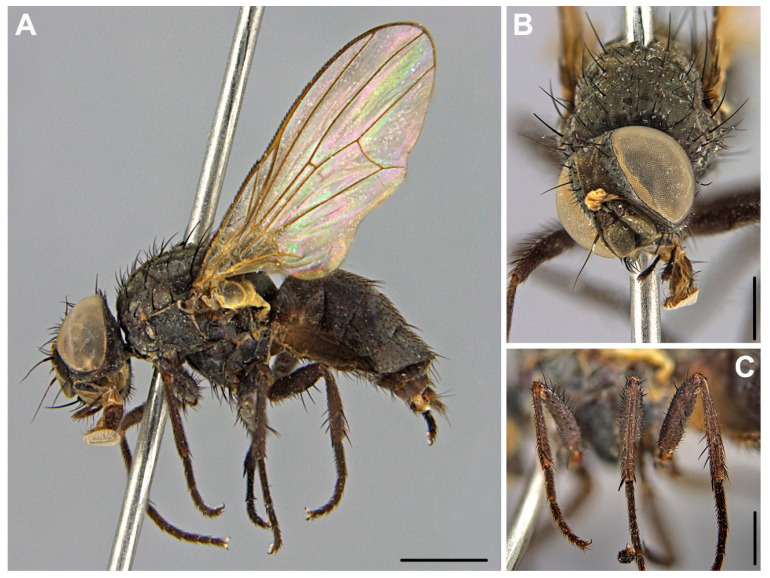
Female of *Fannia collini*: (**A**) lateral view; (**B**) head in anterior view; (**C**) legs in dorsal aspect. Scale bar 1 mm (**A**) and 500 µm (**B**,**C**).

In Figure 5. Material examined. Adult insects: 2♀♀, 29 IV 2012; 1♀, 1 V 2012; 1♀, 6 V 2012; 1♀, 7 V 2012; 1♀, 8 V 2012; 3♀♀, 9 V 2012; 1♀, 13 V 2012; 2♀♀, 14 V 2012; 2♀♀, 15 V 2012; 1♀, 16 V 2012; 2♀♀, 17 V 2012; 5♀♀, 18 V 2012; 9♀♀, 19 V 2012; 5♀♀, 20 V 2012; 5♀♀, 21 V 2012; 1♀, 22 V 2012; 2♀♀, 23 V 2012; 1♀, 24 V 2012; 4♀♀, 26 V 2012; 1♀, 29 V 2012; 1♀, 30 V 2012; 3♀♀, 5 VI 2012; 6♀♀, 7 VI 2012; 6♀♀, 9 VI 2012; 1♀, 13 VI 2012; 3♀♀, 13 VI 2012; 3♀♀, 15 VI 2012; 3♀♀, 28 VI 2012; 1♀, 2 VII 2012; 3♀♀, 6 VII 2012; 2♀♀, 10 VII 2012; 1♀, 22 VII 2012; 1♀, 23 VII 2012; 2♀♀, 24 VII 2012; 3♀♀, 25 VII 2012; 3♀♀, 26 VII 2012; 2♀♀, 27 VII 2012; 1♀, 29 VII 2012; 3♀♀, 31 VII 2012; 3♀♀, 1 VIII 2012; 5♀♀, 2 VIII 2012; 3♀♀, 3 VIII 2012; 3♀♀, 4 VIII 2012; 1♀, 8 VIII 2012; 2♀♀, 10 VIII 2012; 7♀♀, 17 VIII 2012; 1♀, 19 IX 2012; 5♀♀, 20 IX 2012; 2♀♀, 21 IX 2012; 3♀♀, 22 IX 2012; 1♀, 25 IX 2012; 1♀, 27 IX 2012; 1♀, 28 IX 2012; 1♀, 29 IX 2012; 3♀♀, 1 X 2012; 1♀, 2 X 2012; 3♀♀, 8 X 2012; 1♀, 9 X 2012; 1♀, 10 X 2012; 1♀, 14 X 2012; 1♀, 8 V 2013; 1♀, 9 V 2013; 1♀, 15 V 2013; 1♀, 16 V 2013; 1♀, 17 V 2013; 5♀♀, 18 V 2013; 12♀♀, 19 V 2013; 10♀♀, 20 V 2013; 6♀♀, 21 V 2013; 4♀♀, 22 V 2013; 1♀, 23 V 2013; 6♀♀, 24 V 2013; 7♀♀, 25 V 2013; 1♀, 26 V 2013; 12♀♀, 27 V 2013; 6♀♀, 28 V 2013; 1♀, 29 V 2013; 27♀♀, 30 V 2013; 2♀♀, 31 V 2013; 2♀♀, 1 VI 2013; 3♀♀, 2 VI 2013; 37♀♀, 3 VI 2013; 3♀♀, 4 VI 2013; 1♀, 5 VI 2013; 3♀♀, 6 VI 2013; 11♀♀, 7 VI 2013; 2♀♀, 9 VI 2013; 1♀, 11 VI 2013; 2♀♀, 13 VI 2013; 2♀♀, 31 VII 2013; 1♀, 2 VIII 2013; 1♀, 3 VIII 2013; 3♀♀, 6 VIII 2013; 1♀, 7 VIII 2013; 3♀♀, 8 VIII 2013; 4♀♀, 9 VIII 2013; 1♀, 10 VIII 2013; 1♀, 12 VIII 2013; 1♀, 16 VIII 2013; 1♀, 18 VIII 2013; 1♀, 27 VIII 2013; 1♀, 30 VIII 2013; 1♀, 11 IX 2013; 1♀, 23 IX 2013; 1♀, 25 IX 2013; 2♀♀, 1 X 2013; 2♀♀, 5 X 2013; 10♀♀, 6 X 2013; 1♀, 7 X 2013; 2♀♀, 8 X 2013; 1♀, 10 X 2013; 3♀♀, 16 X 2013; 1♀, 17 X 2013; 3♀♀, 21 X 2013; 2♀♀, 22 X 2013; 3♀♀, 24 X 2013; 1♀, 9 XI 2013; hornbeam-oak forest, Biedrusko, Poland, M. Jarmusz leg.

Description of the female.

Body length 4.8 mm, wing length 4.2 mm.

Head. Eyes sparsely haired. Palpi uniform, not flattened, dark, slightly longer than proboscis (Figure 5A,B). Genae, parafacials and fronto-orbital plates grey, frontal vitta brownish in frontal view (Figure 5B) and black in dorsal aspect. Parafacials narrow and bare. Frontal vitta about as broad as fronto-orbital plates. Proboscis dusted. Three pairs of strong and long frontal setae with at least three pairs of shorter setae between them; two pairs of orbital setae. Upper postocular setulae uniserial. Lower orbital setae inserted close to outer margin of fronto-orbital plates. Arista dark, sparsely haired.

Thorax. Ground-color black, greyish dusted without distinct stripes (Figure 5A). Proepisternum bare. Presutural acrostichals biserial, postsutural acrostichals triserial, dorsocentrals 2 + 3. Notopleuron without additional short hairs. Two prealar setae, anterior seta stronger and about half as long as posterior notopleural seta. Proepisternum, meron bare. Calypters yellowish, lower one broad, projecting beyond upper one. Scutellum with two pairs of long setae. Spiracles yellowish.

Wings. Basicosta and veins yellowish-brown. Halters whitish. Wings membrane hyaline.

Legs. Generally dark, except fore tibiae which are narrowly pale at base (Figure 5C). Fore femora with a row of short posterodorsal setae. Fore tibia with only preapical setae. Mid femora apically with row of anterior setae and posterior setae. Mid tibia with 1 anterodorsal and 1 posterodorsal setae. Hind femora with 3 long anteroventral setae in apical half. Inner posterior side of hid coxa with seta. Hind tibia with 1 anterodorsal, 2–3 anteroventral, 1 median and 1 preapical dorsal setae.

Abdomen. Uniformly dark, with gray pruinosity (Figure 5A), without distinct spots or median vitta. Segments 1–3 with indistinct brownish coloration in dorsal view.

## 5. Discussion

### 5.1. Taxonomy

Using DNA barcoding we were able to assign unknown *Fannia* morpho-types to species and reveal their potential forensic utility. However, accuracy of identification by means of DNA barcoding depends on many factors, such as availability of extensive reference library of gene sequences [42]. Taxonomic representation of Fanniidae in reference databases is uneven, with sampling biased towards common species while many other species are underrepresented or represented only by single sequences [10]. Even though undersampling may increase error rate of DNA barcoding [43], in our previous study we observed very high accuracy in species identification using COI barcode sequences in Fanniidae [10]. A distinct barcoding gap was revealed even for species in which females are indistinguishable, e.g., *F. aequilineata* Ringdahl, 1945 and *F. latipalpis* (Stein, 1892) [10]. The misidentification of voucher specimens may be detrimental for end users of reference libraries [42]. However, the identifications we obtained in this study are considered to be valid, as all query sequences had the highest similarity with sequences obtained from what we consider to be accurately identified voucher specimens.

The present state of knowledge on Fanniidae taxonomy is still incomplete. Even though recent studies have improved our taxonomic understanding of this family [44,45], conspecific specimens belonging to different sexes or developmental stages are yet to be described for many species [27]. This gap in knowledge could have severe implications, for example, in Europe where females of many species remain unknown (i.e., *F. alpina* Pont, 1970, *F. brinae* Albuquerque, 1951, *F. conspecta* Rudzinski, 2003, *F. fasciculata* (Loew, 1873), *F. limbata* (Tiensuu, 1938), *F. pseudonorvegica* d’Assis-Fonseca, 1966, *F. rabdionata* Karl, 1940 and *F. ringdahlana* Collin, 1939). This is of particular concern when conspecific males are known to be attracted to carrion and thus by extension are forensically important (i.e., *F. conspecta*) [2,46]. Because of the incompleteness of Fanniidae taxonomy, researchers and practitioners should utilize molecular techniques such as DNA barcoding to verify the identity of specimens which cannot be identified with current keys and/or are discordant with morphological descriptions. The utility of this approach is exemplified in this study, in which molecular barcoding allowed the identification and description of the female of *F. collini*, a species previously known only from males.

Immature stages are still unknown for the great majority of Fanniidae. More than 50 species of fanniids have been reported from decomposing cadavers worldwide [1,2,10]. However, many of these records are based on only a few adult specimens collected from a single cadaver and as such may represent an accidental occurrence rather than a true association. The presence of immature stages can help to confirm a species association with carrion, but this is reliant on the ability to accurately identify these larval stages. The utility of larvae to confirm species association with carrion was exemplified during our two-year carrion succession experiment. Throughout this experiment only two males and two females of *F. nigra* were collected (Jarmusz unpubl.). However, DNA barcoding facilitated association of many unidentified larvae with adult conspecifics, and thus *F. nigra* has been recognized as regular element of necrophagous fauna [16] and as discussed below, a valuable forensic indicator.

The two distinct larval morpho-types which we have examined can easily be placed in the identification key provided by Rozkošný et al. [27], and thus differentiated from remaining European larvae of Fanniidae. First and foremost, the pattern and structure of abdominal dorsolaterals (*dl*) in *F. nigra* and *F. pallitibia* are unique among larvae of Fanniidae. In *F. nigra* thoracic and abdominal *dl* consist of a minutely projected ring, and in *F. pallitibia dl* are completely absent. It should be noted that it is possible that *dl* are present in *F. pallitibia* but in an extremely reduced form and as such we were unable to distinguished them using a stereomicroscope. Nevertheless, in the remaining *Fannia*, even when thoracic *dl* are weakly developed, abdominal *dl* are present as at least minute stalks with a few projections. Only *Euryomma peregrinum* (Meigen, 1826) lacks abdominal *dl*, yet even this species displays *dl* on T2. In addition to the *dl* pattern, *F. nigra* can be easily differentiated from other Fanniidae by the following combination of characters: uniquely small thoracic and abdominal processes, specifically, minute *dm* and *dl*, very short *ap* and *ld* and the latter with only minute lateral projections. Similarly, *F. pallitibia* can be distinguished from other known larvae by the following combination of characters: posterior spiracles placed on lateral margin of the anal division, *dm* minute and filiform, *dl* absent and all processes on the anal division of the same length and equipped basally with long, bifurcated projections.

*Fannia collini* was known only from Great Britain [27,47], until recent collections of this species were reported from Czech Republic (a single male specimen collected in 2012 in Bohemia) [46] and Central Poland (more than 20 males collected during carrion succession experiments; first records originating from 2006) [1]. Even though *F. collini* has been considered a rare European fanniid, we currently recognize it as a regular and relatively frequent element of carrion arthropod assemblages in Central Europe. Females of *Fannia collini* run to the couplet *no 49* in the key for European Fanniidae [27] and key out as *Fannia immutica* Collin, 1939. However, females of *Fannia collini* are morphologically discordant with females of *F. immutica*. A single COI barcode sequence was available for *F. immutica* in the online GenBank database (MF874564), and our molecular analysis showed interspecific distance between *F. immutica* and *F. collini* was higher than 12%. Based on literature data [27,28,47] and examination of specimens deposited in the collection of the Natural History Museum (London, UK), we propose the following combination of characters for the separation of these two species in the key of Rozkošný et al. [27]:

49. Two prealar setae, anterior prealar seta longer than half length of posterior notopleural seta and inserted nearer to suture than to supraalar seta…49a
49a. Hind tibia with 8–10 short anterodorsal setae in addition with 1 strong seta; lower orbital setae in the middle of fronto-orbital plates… *F. immutica* Collin

‒ Hind tibia with 1 strong anterodorsal seta, without additional setae above; lower orbital setae inserted nearer to outer margin of fronto-orbital plates… *F. collini* d’Assis-Fonseca
‒ Only one weak prealar seta inserted nearer to supra-alar seta…50

### 5.2. Forensic Importance

Imprecise identification of material collected during carrion succession experiments gives an incomplete image of necrophagous entomofauna. Comparison of COI barcode sequences against those in depository databases enabled unequivocal identification of fanniids and thus better understanding of the composition of carrion insect assemblages in Central Europe. For this reason, the results we obtained in this study, specifically identifications of *Fannia* sp. 1 as *Fannia nigra*, have already been used in practice and detailed analysis of *F. nigra* association with carrion has been published in Jarmusz et al. [16]. A significant correlation between the appearance time of *F. nigra* third instar larvae and the onset of active decay of carrion decomposition process was revealed by Jarmusz et al. [16]. We confirm this association with data obtained from a case study, that is, third instars of *F. nigra* have been collected from a human body which was in the active stage of decomposition. Recent re-analysis of data provided by Sonet et al. [48] revealed *F. nigra* adult specimen has already been collected from a human cadaver [10], despite the authors not being able to identify it to species level.

Analysis of entomological evidence obtained from the aforementioned casework did not provide unequivocal PMI estimations. According to laboratory observations of Grassberger et al. [39], *Ch. albiceps* larvae in central Europe do not develop below 15 °C. However, Richards et al. [40], after reanalysis of data obtained by Grassberger et al., found that lower developmental threshold for pupariation is T_0_ = 11.65 °C and for eclosion is T_0_ = 10.10 °C. Additional data from other studies confirm *Ch. albiceps* is able to develop in temperatures below 15 °C [49,50]. We used the lower development threshold of *Ch. albiceps* identified by Richards et al. [40] to identify the development time, however, our results were unsatisfactory for the prosecutor in the context of last time the deceased person was seen alive. Estimations from *Ch. albiceps* significantly overestimated, and those from *C. vomitoria* underestimated mPMI.

*Fannia nigra*, was utilized to help refine the calculation of mPMI in this case. According to Jarmusz et al. [16], the appearance time on carrion, (the time until when first specimen of a given taxon was recorded on a carcass) for third instar larvae identified herein as *F. nigra* is 34.8 days in autumn [51]. After analysis of entomological evidence, we conclude that (1) thermal requirements for the development of immature stages of Central European population of *Ch. albiceps* require reinterpretation; (2) the time of cadaver exposure was longer than mPMI estimated from *C. vomitoria* larvae; and (3) PMI estimation obtained from information about *F. nigra* association with carrion were the most congruent with the time the deceased person was last seen alive. In this study we provide, for the first time, morphological data which enable prompt and easy identification of third instars and puparia of *F. nigra* and thus facilitate its broad application as forensic indicator.

Four larvae of *F. pallitibia* have been collected during our carrion succession experiment. As such, *F. pallitibia* was either a rare element of necrophagous fauna or the four larvae were randomly present in the surrounding environment, e.g., in soil beneath the corpse, and their presence was solely an artefact of application of pitfall traps used as part of the collecting protocol. Thus, until future studies investigate this relationship, we refrain from considering *F. pallitibia* as a forensic indicator.

This study revealed, adults of *F. collini* are a regular element of carrion insect assemblages in Central Europe, and in some cases the most abundant fanniid species (data unpublished). As such, we preliminarily assumed immature stages representing *Fannia* sp. 1 or *Fannia* sp. 2 were conspecific with the adult *Fannia* sp. 3 (identified as *F. collini)*, however this was not confirmed. It is possible that, *F. collini* repeatedly visit carrion in adult stage, likely to obtain protein meal, yet its larvae do not develop in decomposing carrion or cadavers. This behavior is commonly observed in other insects [52], e.g., hundreds of adults of *Thricops simplex* (Wiedemann, 1817) and *Pollenia* Robineau-Desvoidy, 1830 have been reported from pig carrion [51,53], however, not a single report of larvae is available for either species. Another, somewhat unlikely, possibility is that larvae of *F. collini* are present on dead bodies, yet despite intensive sampling, they have not been collected. Nevertheless, this study emphasizes the importance of taxonomic skills among forensic entomologists. In particular, a wide knowledge of morphological diversity of adults and immature stages of insects.

## 6. Conclusions

The present study fills a gap in taxonomy of Fanniidae and our knowledge of morphological diversity of the preimaginal instars of *Fannia*. DNA barcoding enabled the assignment of unidentified larvae and females to species and revealed their potential forensic utility. *Fannia nigra* and *F. collini* appeared to be repeatable elements of arthropod carrion assemblages in Central Europe.

## Data Availability

Newly obtained sequences were deposited in GenBank.
